# Comparison of physicochemical properties of different tissues from China climbing perch *Anabas testudineus* and crucian carp *Carassius auratus*

**DOI:** 10.1002/fsn3.2727

**Published:** 2022-01-22

**Authors:** Qiulan Luo, Guangcai Zha, Liyun Lin, Yongping Huang, Xianghui Zou

**Affiliations:** ^1^ School of Life Sciences and Food Engineering Hanshan Normal University Chaozhou China

**Keywords:** amino acids, climbing perch *Anabas testudineus*, crucian carp, freshwater fish, tissues

## Abstract

This study aimed to investigate nutrition in climbing perch *Anabas testudineus* which is an important nutritious economic freshwater fish in Asia and compare with *Carassius auratus* (crucian carp). Three kinds of tissues, including muscle, livers, and eggs, were isolated, respectively. Physicochemical properties including moisture, ash, protein, amino acids, fat, vitamins, and calcium contents in those tissues were determined. The results showed climbing perch muscle and liver contained less moisture, but more protein, amino acids, and vitamins than crucian carp muscle and liver. While moisture, ash, protein, and total amino acids contents of climbing perch egg were lower than those of crucian carp egg. Climbing perch egg had more fat, vitamins, and calcium than crucian carp egg. The amino acid profile was also performed, and 16 amino acids were identified and quantified in muscle, liver, and egg. Among tissues, the highest and lowest concentration of total amino acid content was found in crucian carp eggs and livers, respectively. Glutamic acid (Glu) and histidine (His) were the most and least amino acids in climbing perch and crucian carp tissues, respectively. Sixteen amino acids in climbing perch egg were less than those in crucian carp egg while it is an opposite case in muscle and liver, which amino acids of climbing perch tissues were more than those of crucian carp muscle and liver. Vitamin A of climbing perch was more than crucian carp in all three tissues, but vitamin E content in climbing perch liver was lower than that of crucian carp liver. Calcium content of muscle had no difference between two species. The abovementioned comparison of physicochemical properties of different tissues from China climbing perch and crucian carp will provide a necessary supplementary of freshwater muscle nutrition research, also was helpful for application of climbing perch.

## Graphical abstract

Physicochemical properties of three kinds of tissues, including muscle, livers, and eggs from climbing perch and crucian carp were determined, respectively. The results showed climbing perch muscle and liver contained less moisture, but more protein, amino acids, and vitamins than crucian carp muscle and liver, while moisture, ash, protein, and total amino acids contents of climbing perch egg were lower than those of crucian carp egg. The comparison of nutrition of different tissues from China climbing perch and crucian carp was helpful for application of climbing perch.

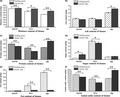

## 1 INTRODUCTION

Food nutrition and security are attracting more and more concerns due to risks of transboundary and emerging animal and zoonotic diseases, such as COVID‐19, foot‐and‐mouth disease, and African swine fever (Cobbold et al., 2021). In past decades, aquaculture is one of the fastest growing food producing sectors, because aquatic fish provides an increasing proportion of high value food for humans (Béné et al., 2015). Compared with nutrient composition of terrestrial farmed meat products, aquatic animal foods represent more nourishment and healthy. Aquatic animal foods contain more protein and moisture, better essential amino acid (EAA) profile, less saturated fat, and the highest concentration of long‐chain omega‐3 [(*n*‐3)] polyunsaturated fatty acids of any foodstuffs, including eicosapentaenoic acid (EPA) and docosahexaenoic acid (DHA) (Tacon & Metian, 2013). Aquaculture and fisheries are both crucial to solve the short‐term need for food aid to deal with the world's hunger and malnutrition problems. Fish has been the major animal source food in many low‐ and middle‐income countries such as South Asia and sub‐Saharan Africa yet, where population increased quickly, and agricultural land was limited for sustainable food production (Lipper et al., 2014). At the same time, fish could be captured or cultured without land, and fish products were developed rapidly due to its day by day increasing demands.

Large amount of fish was consumed every year in China. As one of the major fishery countries in the world, China produced plenty of fish to fulfill both domestic use and export demand. In 2018, China exported exceeded 50 million tons aquaculture to other countries or regions, which accounts for more than 78% of the total output of aquatic products in the world (MOA, 2021). Also, China was the only country in the world where the output of aquaculture aquatic products exceeds that of fishing. Although fisheries and aquaculture sector had declined slightly in 2019 due to impacts of the COVID‐19 outbreak, it did not change the fact that fish is the main food and important source of nutrition for Chinese people (China Bureau of Fisheries, 2021). However, the species of farmed freshwater fish need more diversity. The data showed in 2010, that nine kinds of freshwater fish species made up 86 percent of fish supplied by aquaculture (Tacon & Metian, 2013). Among the farmed freshwater fish, crucian carp (*Carassius carassius*) takes a large proportion in aquaculture. Here, we studied a wild freshwater fish climbing perch *Anabas testudineus*, and compared with crucian carp, in order to develop one more kind of farmed fish species.

*Anabas testudineus* commonly known as climbing perch or koi, is an economic freshwater fish which widely distributes throughout south and south‐east Asia. Although climbing perch is very hardy, its meat has delicate quality, delicious taste and high price, so it has occupied a large freshwater muscle market share in south Asia recently. Climbing perch is considered as healthy diet for sick and convalescents, due to its good source of proteins, plenty of essential amino acids, easily digestible fat, and bio‐nutritionally available mineral elements such as iron and copper that support hemoglobin synthesis (Hossain et al., 2015). It was also reported to have medicinal properties such as disease prevention and anti‐aging process for females. Meanwhile, climbing perch is an air‐breathing teleost that can withstand wide environmental conditions such as low oxygen, wide range of temperature and salinity, and markedly contaminated and other poor water conditions (Chew & Ip, 2014). The wide adaptability indicated climbing perch is a potential good quality freshwater cultivated species. The geographic distribution of climbing perch is also very wide including swamps, lakes, reservoirs, canals, pools, rice fields, rivers small pits, and puddles. It has generated much interest in aquaculture production in many tropical Asian countries and regions, including India, Bangladesh, Sri Lanka, Burma, southern China, and Malaysia, because of its high demand, superior flesh quality, and high market price (Kohinoor et al., 2009). Besides, this fish is studied as an excellent ecosystem model to understand the divergence in various traits.

Since 2001, climbing perch has been considered as one of the potential new candidate species for aquaculture and captive breeding. After developing for decades in Asian, scientists have made certain progress in the feeding of climbing perch. There were several reports about ontogenesis, reproductive ecology and cultivation of climbing perch (Pavlov, 2021; Sarkar et al., 2005). Breeding climbing perch by spawned eggs using hormonal induction and farming fry fish in brackish water in different salinity were performed successfully (Chotipuntu & Avakul, 2010). Recent reports referred that the nutrition of egg and muscle is bound up with climbing perch fish production, the improvement of diet of adult climbing perch, could increase its antioxidants and protein contents (Manju et al., 2012). Liver is an important organ for climbing perch to adapt various environments. Transcriptomic analysis of climbing perch exposed under pesticides showed genes in liver markedly altered, indicating the liver is important toxic elimination tissue for climbing perch (Zhang et al., 2019). The components properties of liver and egg of climbing perch which were detected in this study can provide a better understanding on this species. By now, reports about nutrition of climbing perch tissues especially China climbing perch were still limited. To learn about this important food fish, the nutrient profile of China climbing perch muscle, liver, and egg was performed to reveal the nutrition of this wild species. Moreover, compared with crucian carp, which is one of freshwater fish popular in China, highlights of climbing perch nutritive values, will be helpful for extending the application of climbing perch.

## 2 METHODS

### 2.1 Fish samples

Adult climbing perch wild fish with body weights varied from 53.2 to 90.1 g were captured from local rivers in Chaozhou, China (23°26′‐24°14′ N, 116°22′‐117°11′ E). Besides, crucian carp (800 ± 50 g) were purchased from the local market of Chaozhou, China. After cut and deboned, the fish tissues such as muscle, livers, and eggs were separated and collected. All samples were stored at −20°C until analysis (The maximum storage time is 3 months).

### 2.2 Moisture and ash content analysis

Moisture content of samples was determined by “dried” method (AOAC, 2005). Approximately, 2.0 g of sample was grinded and dried in ovens (Lianjing, Nanjing, China) at 105°C for 4 h and then weighed. The drying and weighing were continued until the difference between two consecutive weightings was within 2 mg. Measurements of each sample were repeated three times. Ash content of fresh samples was measured using incineration method at 550°C by Muffle Furnace (Duchuang, Beijing, China). The results were presented as percentage of wet weight basis.

### 2.3 Protein, fat, and sugar content determination

The crude protein content was examined using Kjeldahl method (AOAC, 2005, Method No. 981.10) and 6.25 was used as conversion factor, as described previously (Valverde et al., 2012). The total fat content was determined using by acid‐hydrolysis method (Lakshanasomya et al., 2011). Briefly, fat was degraded with 2 M hydrochloride, then dried and extracted by Soxhlet extractor (Ouge, Shanghai, China), finally weighed. The soluble sugar content was detected via photometric method (Dubois et al., 1956) using a calibration curve, which was constructed by using the glucose solution. The solution sugar in samples was extracted in a water bath (100°C) for 30 min, after cooling and centrifuged, 1 ml of 5% phenol reagent and 10 ml concentrated H_2_SO_4_ was added to the supernatant, then vigorously vortex and detected at a wavelength of 470 nm using an ultraviolet spectrophotometer (Aoe). Protein, fat, and sugar contents were expressed as gram of equivalents for per kilogram fresh weight.

### 2.4 Analytical methods and determination of amino acids

Before amino acids chemical analyses, crude protein needs to be determined by the Kjeldahl method (Valverde et al., 2012). Amino acids were determined according to the method GB5009.124–2016 in China. Frozen samples containing 10–20 mg protein were used and prepared as tissue homogenates by grounding. For acid digestion, sample was mixed with 6 M hydrochloride and refrigerant in a screw‐cap tube and cooled for 5 min. Tubes were hooked up by the air exhaust pipe of vacuum pump, then vacuumed and nitrogen‐filled for three time, and incubated at 110°C ± 1°C for 22 h. The samples were cooled at room temperature, and washed a small number of times with 50 ml water. Then 1 ml solution was introduced into 15 ml tubes, and dried at 50°C by a vacuum concentrator (Organomation Associates). The dried product was diluted in 2.0 ml of 0.2 M sodium citrate buffer (pH 2.2), and filtered into a 2 ml screw vial using 0.22 µm PVDF syringe filters. Finally, amino acids of the samples were measured by L‐8900 type Amino Acid Automatism Analyzer (Hitachi) followed the external standard method. Each of 16 amino acids being tested was weighed exactly, mixed and diluted with 6 M hydrochloride to make a 1 mM standard stock solution. Before used, amino acids standard solution was diluted 10 times by 0.2 M sodium citrate. The content of each amino acid was calculated according to the Equation (1).

(1) $$X_{i}=c_{s}\times A_{i}\times F\times V\times M/A_{s}\times m\times 10^{8}$$

where, *X_i_* = each amino acid content of sample (g/kg); *A_i_*, *A_s_* = the peak area of amino acid of sample and standard amino acid, respectively; *F* = dilution rate; *V* = volume of transferred solution (ml); *M* = molecular weight of each amino acid; *m* = weight of sample (g). All samples were tested in triplicates.

### 2.5 Determination of calcium (Ca) content

Calcium (Ca) in samples was examined according to the method as described previously (Harrington et al., 2014), with some modifications. In brief, about 1.0 g samples were prepared as tissue homogenate by grinding, then wet digested with 6 ml HNO_3_ and 2ml H_2_O_2_ (30%) at room temperature for 12 h and subsequently at 100°C for 5 h in microwave. After cooled, the digested products were acid‐driving at 140°C for 30 min, and then diluted to 25 ml by water. The sample was diluted 1000 times, filtered and measured by Inductively Coupled Plasma Optical Emission Spectrometer (ICP‐OES, PerkinElmer). The Ca content (mg/kg) was calculated based on Ca standard curve.

### 2.6 Vitamin A and E content examination

Vitamin A and E examination was performed by the method modified from Scalia et al. (2010). About 5.0 g of sample was crushed and accurately weighed into a 150 ml beaker, with 20ml of warm water (40°C) and 1 g of amylase added. The mixture was stirred to dissolve the powder at 60°C for 30 min. For saponification, 1.0 g ascorbic acid, 0.1 g butylated hydroxytoluene (BHT), 30 ml ethanol and 20 ml of 50% potassium hydroxide were added with in the matrix. After stirring, and the mixture was bathed at 80°C for 30min, and cooled to room temperature with cold water. The saponified vitamin A and E were extracted using petroleum ether–diethyl ether (1:1) reagent. The under‐layer solution was collected, filtrated, concentrated, dried by anhydrous sodium sulphate, and then analyzed by high‐performance liquid chromatograph (HPLC) system (Shimadzu, Kyoto, Japan).

### 2.7 Statistical analysis

All experiments were conducted in triplicate. The results are presented as the mean value ±the standard deviation. The differences between the groups were calculated statistically using SPSS 20.0 software. Significance was defined at *p* < .05 using analysis of variance (ANOVA).

## 3 RESULTS

### 3.1 Proximate composition of climbing perch and crucian carp tissues

**FIGURE 1:**
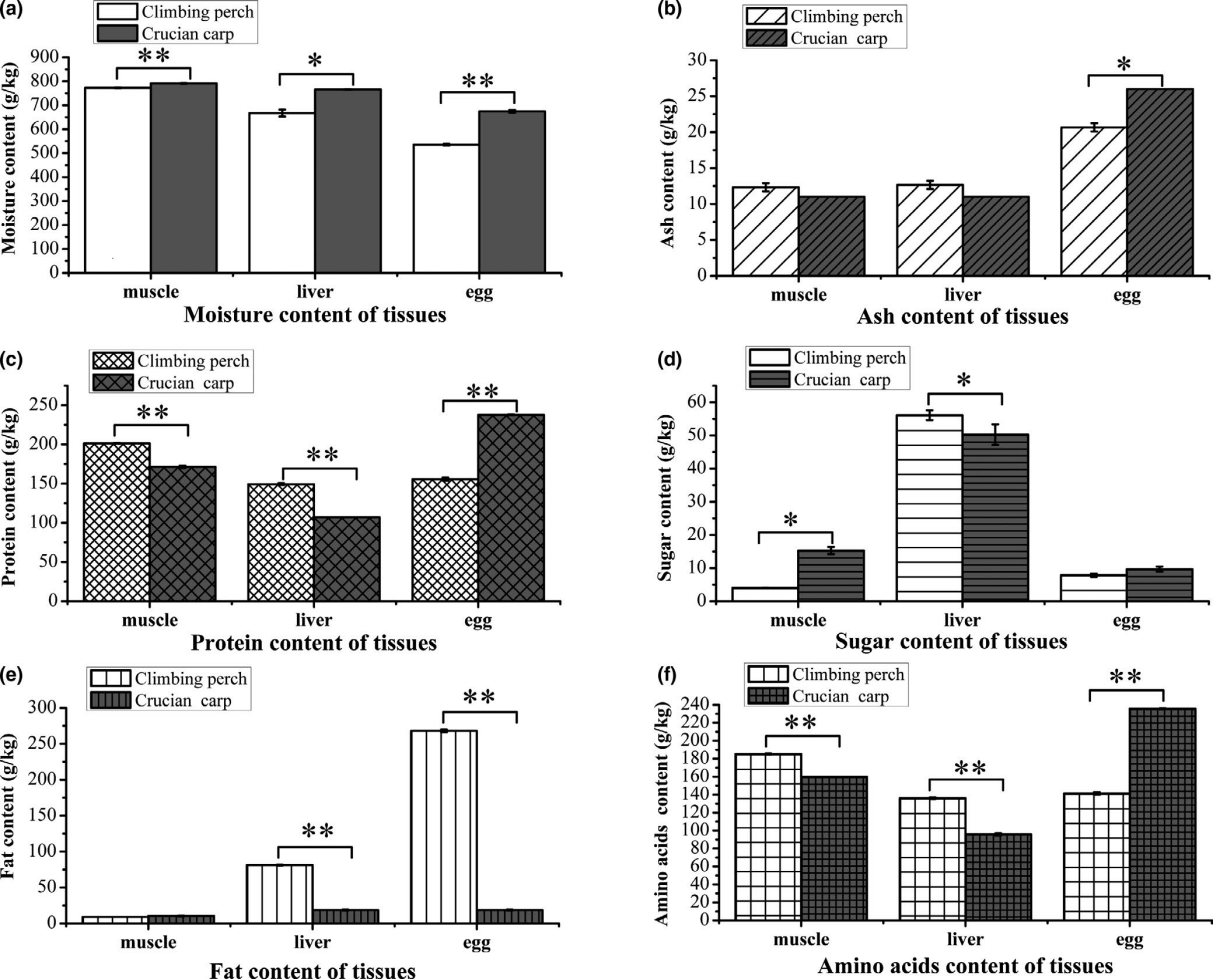
Proximate composition of climbing perch and crucian carp tissues, including (a) moisture, (b) ash, (c) protein, (d) sugar, (e) fat, and (f) amino acids content in egg, liver and muscle. Bars represent mean ± *SD*. Asterisk * and ** indicate a significant difference *p* < .05 or *p* < .01 from each other, respectively

After quantitative measurement, the composition properties of tissues, containing muscle, liver, and egg, which were from climbing perch and crucian carp were compared. As shown in Figure 1, tissues in climbing perch and crucian carp were different in moisture, ash, protein, sugar, fat, and amino acids contents. Moisture content of climbing perch showed a lower level than that of crucian carp, down by 2.40%, 12.88%, and 20.56% in muscle, liver, and egg, respectively (Figure 1a). Besides, the ash content in climbing perch and crucian carp tissues seemed similar without egg, in which ash content of climbing perch was 20.51% less than that from crucian carp (Figure 1b). The sugar content of climbing perch and crucian carp tissues showed muscle and liver presented difference, which in climbing perch muscle 73.64% less and 1.1‐folds more in liver than that in crucian carp' s (Figure 1d). The protein and amino acids content of all three tissues of climbing perch and crucian carp showed the same tendencies (Figure 1c,f). Protein contents of climbing perch muscle, liver, and egg were 201.3 g/kg, 149.3 g/kg, and 155.7 g/kg, respectively; compared with crucian carp samples this equates to increase of 39.56% and 17.51%, and decrease of 34.5%, respectively. On the other hand, total amino acids contents in egg of climbing perch were 40.03% less than that of crucian carp egg. While amino acids contents of climbing perch liver and muscle were 41.91% and 15.83% more than those of crucian carp liver and muscle. And as shown in Figure 1e, the amount of fat in climbing perch egg and liver was much more than that of crucian carp egg and liver, with a multiple of 14.36 and 4.36 times, respectively.

### 3.2 Amino acids profile of climbing perch and crucian carp different tissues

As shown in Table 1, 16 types of amino acids were identified and quantified in the climbing perch and crucian carp muscle, liver and egg, which were aspartic acid (Asp), threonine (Thr), serine (Ser), glutamic acid (Glu), glycine (Gly), alanine (Ala), valine (Val), methionine (Met), isoleucine (Ile), leucine (Leu), tyrosine (Tyr), phenylalanine (Phe), lysine (Lys), histidine (His), argnine (Arg), and proline (Pro). Among these amino acids, Glu has the highest content in each type of tissue, ranged from 12.50 g/kg (crucian carp liver) to 37.63 g/kg (crucian carp egg). And His was the least amino acid in climbing perch and crucian carp tissues. Compared egg of climbing perch and crucian carp, amino acids showed significant differences at *p* < .05. Climbing perch egg contains lower amino acids than crucian carp egg, with declined degree from 33.52% (Gly) to 77.38% (Met). In contrast, amino acids content in climbing perch liver was much higher than that of crucian carp liver, which increased by 1.26 time (Arg) to 1.53 time (Tyr). However, Ser, His, and Pro contents were found to exhibit no difference in muscle amino acids of climbing perch and crucian carp. Other 14 amino acids were abundance in climbing perch muscle, which varied from 5.73 g/kg (Met) to 29.00 g/kg (Glu). Besides, these amino acids contents of climbing perch muscle were higher than 0.83 g/kg to 4.33 g/kg, that about crucian carp muscle. Moreover, the amino acid profile showed Asp, Leu, and Lys varied significantly across the different tissues.

**TABLE 1:** Amino acids contents (g/kg) of climbing perch and crucian carp tissues

Amino acids	Climbing perch			Crucian carp		
	Muscle	Liver	Egg	Muscle	Liver	Egg
Essential amino acids (EAA)						
Arg	11.67 ± 0.06^a^	8.07 ± 0.06^b^	9.87 ± 0.06^c^	9.90 ± 0.10^c^	6.40 ± 0.0^d^	14.00 ± 0.10^e^
His	4.43 ± 0.06^a^	4.07 ± 0.06^b^	3.87 ± 0.06^b^	5.23 ± 0.06^c^	2.80 ± 0.0^d^	5.63 ± 0.06^c^
Ile	8.80 ± 0.0^a^	6.47 ± 0.06^b^	7.67 ± 0.21^c^	7.53 ± 0.06^c^	4.73 ± 0.06^d^	11.67 ± 0.21^e^
Leu	16.33 ± 0.06^a^	12.63 ± 0.06^b^	12.10 ± 0.0^c^	13.90 ± 0.10^d^	8.70 ± 0.10^e^	21.23 ± 0.12^f^
Lys	18.27 ± 0.06^a^	11.60 ± 0.10^b^	11.07 ± 0.12^c^	16.03 ± 0.12^d^	8.53 ± 0.06^e^	15.43 ± 0.15^f^
Met	5.73 ± 0.06^a^	3.80 ± 0.0^b^	4.33 ± 0.06^b^	4.90 ± 0.0^b^	2.70 ± 0.10^c^	5.60 ± 0.0^a^
Phe	8.23 ± 0.06^a^	6.63 ± 0.06^b^	6.60 ± 0.10^b^	7.50 ± 0.0^c^	4.73 ± 0.06^d^	8.93 ± 0.06^e^
Thr	8.80 ± 0.0^a^	7.37 ± 0.06^b^	7.13 ± 0.06^b^	7.60 ± 0.04^c^	4.97 ± 0.12^d^	11.50 ± 0.10^e^
Val	9.63 ± 0.06^a^	8.90 ± 0.10^b^	8.87 ± 0.21^b^	8.40 ± 0.0^b^	5.97 ± 0.06^c^	14.20 ± 0.17^d^
∑EAA	91.90 ± 0.30^a^	69.53 ± 0.31^b^	71.50 ± 0.61^b^	81.00 ± 0.23^c^	49.53 ± 0.49^d^	108.20 ± 0.75^e^
Non‐essential amino acids (NEAA)						
Asp	20.07 ± 0.06^a^	13.57 ± 0.15^b^	12.20 ± 0.10^c^	17.67 ± 0.06^d^	9.67 ± 0.21^e^	17.00 ± 0.10^f^
Gly	11.13 ± 0.06^a^	7.73 ± 0.15^b^	6.03 ± 0.25^c^	8.37 ± 0.38^b^	5.33 ± 0.06^c^	18.00 ± 0.20^d^
Ala	12.60 ± 0.0^a^	9.13 ± 0.15^b^	12.50 ± 0.30^a^	10.70 ± 0.10^c^	6.13 ± 0.06^d^	18.20 ± 0.17^e^
Ser	7.27 ± 0.32^a^	6.43 ± 0.29^a^	8.90 ± 0.10^b^	6.47 ± 0.15^a^	4.60 ± 0.10^c^	14.90 ± 0.17^d^
Glu	29.00 ± 0.26^a^	17.70 ± 0.17^b^	17.20 ± 0.0^b^	24.67 ± 0.32^c^	12.50 ± 0.20^d^	37.63 ± 0.12^e^
Pro	6.47 ± 0.49^a^	6.17 ± 0.06^a^	6.77 ± 0.21^a^	5.50 ± 0.17^a^	4.17 ± 0.06^b^	12.73 ± 0.06^c^
Tyr	6.53 ± 0.06^a^	5.90 ± 0.0^b^	6.13 ± 0.15^ab^	5.60 ± 0.10^b^	3.87 ± 0.06^c^	9.27 ± 0.06^d^
∑NEAA	66.63 ± 0.40^a^	127.73 ± 0.55^b^	69.73 ± 0.55^a^	78.97 ± 0.06^c^	93.07 ± 1.01^d^	46.27 ± 0.70^e^
EAA/NEAA	1.04^a^	0.85^b^	1.03^a^	1.03^a^	0.99^a^	1.07^a^

Note

Data are mean ± *SD* of triplicate determinations. Different superscript letters in the same column significantly different (*p* < .05). ∑EAA and ∑NEAA indicated the total essential and non‐essential amino acid content, respectively.

The different letters in the same column just show significantly different, didn’t have other means.

### 3.3 Calcium content of climbing perch and crucian carp tissues

**FIGURE 2:**
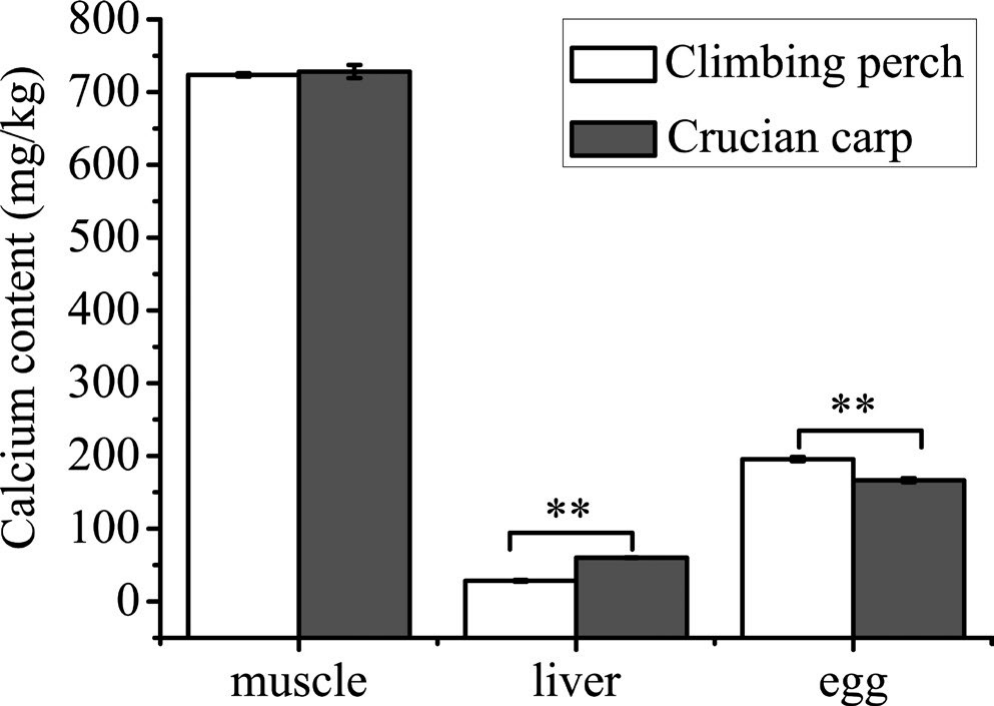
Calcium content of climbing perch and crucian carp tissues. Bars represent mean ± *SD*. Asterisk ** indicate a significant difference *p* < .01 from each other, respectively

The quantitative analysis of calcium (Ca) in three tissues of climbing perch and crucian carp is presented in Figure 2. Calcium in muscle showed the highest concentration compared to other tissues with values of 723.67 and 728.33 mg/kg in climbing perch and crucian carp, respectively. Ca concentration of climbing perch egg was significantly higher than that of crucian carp egg (*p* < .01), about 1.17 times. However, it is an altogether different situation of Ca content in liver, which climbing perch liver had 52.85% less than that of crucian carp liver.

### 3.4 Vitamins in climbing perch and crucian carp tissues

The vitamins including vitamin A, α‐ Vitamin E, and γ‐ Vitamin E were examined through HPLC. As shown in Table 2, the content of vitamin A of climbing perch tissues varied from 2.90 mg/kg to 374.67 mg/kg. Whereas the content of vitamin A of crucian carp egg and liver was 0.28 mg/kg and 4.18 mg/kg, respectively, and none of vitamin A was detected in crucian carp muscle. Vitamin A of climbing perch egg was 10.36 times more than that of crucian carp egg, while in liver that increased to 89.63 times difference. Two kinds of vitamin E, α‐vitamin E and γ‐ vitamin E were also examined. However, none of α‐vitamin E was detected in climbing perch liver, and climbing perch had more α‐vitamin E than crucian carp in egg and muscle. By the way, γ‐ Vitamin E was only detected in crucian carp egg with a concentration of 11.87 mg/kg.

**TABLE 2:** Vitamins contents (mg/kg) of climbing perch and crucian carp tissues

Vitamin	Climbing perch			Crucian carp		
	Muscle	Liver	Egg	Muscle	Liver	Egg
Vitamin A	246.67 ± 5.51^a^	374.67 ± 82.66^b^	2.90 ± 0.42^c^	NA	4.18 ± 0.24^d^	0.28 ± 0.01^e^
α‐Vitamin E	5.24 ± 0.03^a^	NA	46.77 ± 2.08^b^	2.25 ± 0.02^c^	6.65 ± 0.19^d^	18.87 ± 0.45^e^
γ‐Vitamin E	NA	NA	NA	NA	NA	11.87 ± 1.86

Note

Data are mean ± *SD* of triplicate determinations. NA indicates data not available. Different superscript letters in the same column significantly different (*p* < .05).

The different letters in the same column just show significantly different, didn’t have other means.

## 4 DISCUSSION

It is widely known that fish and fish products have rich nutritional values, especially their high contents of proteins with essential amino acids (EAAs). Recently, researchers found fish protein hydrolysate had various biological activities and functional properties, and thus could be applied as a human health‐promoting ingredient (Nirmal et al., 2021). Due to high protein content, climbing perch has been assumed to be of high dietary quality and popular in south Asia. As a typically small inland capture fishery, the edible parts of climbing perch are often considered as whole fish without bones, viscera, fins, scales, and gills. In this study, the nutrition including proteins, fats, sugar, amino acids, vitamins, and calcium were detected abundance in climbing perch muscle which is the major edible part. In comparison with Bangladesh and Indian climbing perch, muscle of climbing perch used in this study had much more proteins (201.3 g/kg), but less fats (9.0 g/kg), and similar moisture contents (Figure 1; Bogard et al., 2017; Paul et al., 2017). Fish as one of important protein source foods for human, its protein has higher quality and more digestibility compared to other animal source foods such as eggs, milk, and meat, so fish protein content attracted a lot of attention by decades. According to the estimation, the protein content of fish muscle ranges between 16% and 20% (Gopal, 2020), here the muscle of China climbing perch contained 20.1% protein (Figure 1c), indicating it may be a good kind of protein sourced food. Besides, in recent years, the fish protein extracted from byproducts comprising skin, head, viscera, trimmings, liver, frames, bones, and roes is another important supplement of protein diets in developing countries (Chalamaiah et al., 2012). In this study, high protein concentrations of 149.3 g/kg and 155.7 g/kg also have been detected in the liver and egg of climbing perch, respectively (Figure 1c), showing the by‐products of climbing perch containing rich protein which could be useful in fish food industry.

So far, the fish protein hydrolysates have attracted a lot of concerns due to their absorbable peptides and amino acids for gut microbiota (Nirmal et al., 2021). Nutrition research showed essential amino acids (EAA) have benefits for human growth and development. The amino acid composition of the protein is crucial for determining its hypocholesterolemic properties. All essential amino acids (EAA) which was recommended for adult daily consumption by WHO/FDA (2007) were found in this study (Table 1). This study also denoted that EAAs contents of climbing perch muscle were higher than that of the crucian carp (Figure 1f) and edible portion of Indian climbing perch as reported by Paul et al. (2017). Glutamine has been considered as one kind of main amino acid in fish, has many physiologic function in organs (Christina et al., 1999). In this study, Glutamine was the most abundance amino acids in climbing perch (Table 1), and had a concentration of 29 g/kg in muscle which was higher than that of south Asia climbing perch (Bogard et al., 2015). For other amino acids, although His as an important nutritional amino acid for adults was limited in climbing perch, Lysine another required amino acid for human being was rich in muscle. Fish has balanced essential/non‐essential amino acids ratio (Gopal, 2020). Here, almost equal amount of essential and non‐essential amino acids was detected in muscle of climbing perch (Table 1), showing China climbing perch has applied potential as high‐quality protein source. Furthermore, protein hydrolysates (PH) derived from fish eggs were reported to have different proximate composition, functional properties, and molecular weight with other fish‐source PH (Chalamaiah et al., 2010). Studies showed that carp egg PH significantly increased the splenic NK cell cytotoxicity, mucosal immunity (S‐IgA) in the gut and level of serum immunoglobulin A (IgA) in BALB/c mice (Chalamaiah et al., 2015). Although the amount of protein and total amino acids in eggs of climbing perch were lower than that in crucian carp (Figure 1 and Table 1), also less than that reported for Nile tilapia, another important species of cultured freshwater fish (Gunasekera et al., 1996), its amino acid composition was similar to the carp eggs. The kinds of major amino acids in climbing perch eggs were analogous to that of trout cod and Murray cod (Gunasekera et al., 1999). So, in the views of nutrition, China climbing perch used in this study is a good animal protein source. However, the protein properties of climbing perch need to be further discovered, especially their biological functions.

High‐quality fat is another important nutrition that fish supplies for human, because of its various biological functions, such as prevent parasite infection, tumor initiation, and inflammation (Tallima & Ridi, 2018). At present, fat content of China climbing perch muscle was as high as that of crucian carp (Figure 1e). But fat concentration of climbing perch was 9.0 g/kg, which is lower than other China freshwater fish that ranged from 17.1 g/kg in swamp eel to 74.3 g/kg in black carp (Li et al., 2011). Somehow, more fat did not mean better for human health, and the fatty acid composition should be more concerned. Therefore, the fatty acids composition and functions of from China climbing perch needs further identification in future. By the same time, a comparison was made in climbing perch from different areas such as China, Bangladesh, Indian, and Thailand, their fat contents presented a regional variety which may be caused by different fat storage capability in seasonal/life cycle variations and the various diet/food supplements.

It is well documented that higher protein intake generally leads to a higher calcium intake. Calcium is an essential element for human especially for bone health (WHO, 2007). The data showed intake of dietary calcium is low in developing countries also including China (Hu et al., 1993). It has been attracting much attention to increase intakes of calcium among children and women of childbearing age. Climbing perch is one kind of small fish, which eaten with bones, is also a dietary source of highly bioavailable calcium. This study showed that calcium content in climbing perch muscle was as many as that of crucian carp, with a high value of 723.7 mg/kg (Figure 2). The eggs another edible part of climbing perch also contained abundance of calcium (Figure 2). All these results indicate that the application of climbing perch will be benefit for calcium absorption of China population. Previous studies showed small fish is an important dietary source of both vitamin A and calcium in developing countries (Roos et al., 2003). Vitamin A plays an essential role in human immune function, growth, and vision, and is also necessary for fish growth (Kwasek et al., 2020). Deficiency of vitamin A may cause prevalence of clinical symptoms in child, like night blindness. This health problem could be solved by Vitamin A intakes from diets. High concentration of vitamin A was detected not only in climbing perch muscle, but also in liver and eggs with hundreds of times more than that of crucian carp (Table 2). The results suggested climbing perch may be suitable for being developed into a vitamin A product source for human. Moreover, α‐Vitamin E, another important vitamin, was shown to be abundance in climbing perch muscle and egg, but not in liver (Table 2). Vitamin E often functions in anti‐aging, immunity, and antioxidant through scavenging lipid peroxyl radicals and preventing the propagation of lipid peroxidation (Wang & Quinn, 2000). Here the distribution of Vitamin E is similar as reported earlier in Indian climbing perch (Paul et al., 2017). It is interesting that γ‐ vitamin E was hardly found in crucian carp egg in this study; the possible reason is that γ‐ vitamin E was mainly originated from plant sources but not animals as reported by Jiang et al (Jiang et al., 2001). Given all of that, in the views of nutrition, the climbing perch has its own competition advantage.

Moreover, for decades, most studies have demonstrated growth performance of climbing perch (Chotipuntu & Avakul, 2010), since it has a wide range of adaptation, for instance, the culture temperature, the salinity, and contamination. As we know, egg is closely related to fish species fecundity, which is an important index for fish feeding, while liver is an important detoxifying organ for fish to survive widely. From the properties of eggs and liver, we could preliminary understand why it could survive in variety conditions. The high fecundity of climbing perch is security of fish population. A close connection between fish muscle growth rates and egg states has been revealed recently, that eggs incubation influenced climbing perch growth performances (Ahammad et al., 2021). Here, the size and ash weight of climbing perch eggs was much less than that of crucian carp eggs (Figure 1b), which may explain why the body of climbing perch is much smaller than crucian carp. The abundant fat observed in the eggs of climbing perch, significantly higher than the value detected in eggs of crucian carp (Figure 1f), which may be one reason of the wide adaptation of climbing perch. Proteins in fish eggs are necessary for embryonic and larval development, which could be degraded to amino acids for energy production until first feeding (Hou & Fuiman, 2019). Amino acid composition of eggs of many kinds of freshwater fish species was related to fertilizability and hatchability. At present, the qualitative of amino acids showed the predominant essential amino acids in climbing perch and crucian carp eggs were arginine, lysine, leucine, and valine, indicating that there is a certain particular profile in the essential amino acids of freshwater fish eggs, making them to inhabit and reproduce in freshwater. In addition, one feature of climbing perch is its ability to utilize liver glycogen to build up muscle glycogen and blood glucose under lindane intoxication (Bakthavathsalam & Reddy, 1983), which explained why more sugar was contained in climbing perch liver (Figure 1d). Further the accumulation of sugar in climbing perch liver might be stored as energy source against poor environment. Among detected 16 kinds of amino acids, climbing perch liver contained much more amino acids than crucian carp liver (Table 1). Moreover, the data that liver fat of climbing perch were significantly more than that of crucian carp (Figure 1e) proved that bottom dwelling fish species usually stored fat in the liver. In livers, the free fatty acids were combined with plasma serum proteins, fats were also transported as form of lipoprotein, and proteins play important roles in fat metabolic and transportation. The accumulation of both fat and proteins in climbing perch may be indicating more vigorous fat metabolism in climbing perch liver. All these results indicate climbing perch biosynthesize plenty of balanced biological compounds including sugars, fat, proteins, and amino acids in eggs and liver, making it more adaptive to environment and potential aqua‐cultured.

In conclusion, climbing perch muscle contained abundance proteins, balance amino acids and vitamins, which indicating climbing perch is likely to be a dietary resource food to meet consumers demanding for high‐quality fish products. Egg of climbing perch comprised high concentration of fat, vitamins, and calcium, while liver was rich in sugar, proteins, amino acids, and vitamin A, ensured climbing perch wildly adaptability. So, climbing perch may be a good market species in future.

## CONFLICT OF INTEREST

The authors declare no conflict of interest.

## ACKNOWLEDGEMENTS

We thank Dr. Kang Xao for the comments and corrections of English.

## CONSENT TO PARTICIPATE

All authors have read and approved the content and agreed to submit for consideration for publication in the journal.

## Data Availability

The data and materials are available.
